# Advanced moderately differentiated neuroendocrine carcinoma of the rectum with favorable prognosis by postoperative chemoradiation

**DOI:** 10.1186/1477-7819-8-29

**Published:** 2010-04-17

**Authors:** Hiroyuki Nojima, Kazuhiro Seike, Chihiro Kosugi, Takashi Shida, Keiji Koda, Kenji Oda, Shigeyuki Kamata, Hiroshi Ishikura, Masaru Miyazaki

**Affiliations:** 1 Department of General Surgery, Graduate School of Medicine, Chiba University, Chiba, Japan; 2 Department of Molecular Pathology, Graduate School of Medicine, Chiba University, Chiba, Japan

## Abstract

Rectal neuroendocrine carcinoma is rare with poor prognosis. We report herein a case of advanced moderately differentiated neuroendocrine carcinoma of the rectum with relatively favorable prognosis treated by postoperative adjuvant chemoradiation therapy. A 58-year-old Japanese female was referred and colonofiberscopy revealed an easy-bleeding irregular tumor in the lower rectum, which was pathologically diagnosed as a neuroendocrine carcinoma. Surgical treatment consisted of abdominoperineal resection and lymph node dissection. The tumor invaded deeply into perirectal tissues, and 9 of 11 lymph node metastases were observed. Immunohistochemically, chromogranin A showed diffuse and strong staining, and the MIB-1 labeling index was 18.3 ± 5.6, supporting the high proliferation of the tumor. Some nucleus of the tumor showed positive staining for p21/WAF1. A total dose of 46 Gy of radiotherapy was delivered with 800 mg of daily oral doxifluridine. At 5 years post-surgery, the patient demonstrated no clinical evidence of intrapelvic recurrence or distant metastases.

## Background

Neuroendocrine carcinomas of the colon and rectum are rare tumors with aggressive behavior and poorer prognosis compared with adenocarcinomas, and the reported 3-year survival rates are 13-15%[1]. These carcinomas are subclassified into two pathological types, small cell carcinomas and moderately differentiated neuroendocrine carcinomas. Small cell carcinoma of the colon and rectum is virtually indistinguishable from small cell lung cancer morphologically and immunohistochemically, and small cell lung carcinoma is sensitive to chemotherapy and adjuvant chemotherapy after surgery results in prolonged survival[2]. Several studies have demonstrated the efficacy of chemotherapy for colorectal small cell carcinoma[3]. On the other hand, moderately differentiated neuroendocrine carcinoma of the colon and rectum has a similar morphology to large cell lung carcinoma with neuroendocrine features. Surgery is a mainstay of beneficial treatment although the effect of adjuvant treatment remains undermined.

We herein report a case of advanced moderately differentiated neuroendocrine carcinoma of the rectum with relatively favorable prognosis by postoperative adjuvant chemoradiation therapy.

## Case Presentation

### Clinical history

A 58-year-old Japanese female was admitted to hospital with a two-month history of rectal bleeding. Colonofiberscopy revealed a tumor in the lower rectum, however, a biopsied specimen from the tumor showed no malignant findings. She was referred to our institution for further examinations.

**Figure 1:**
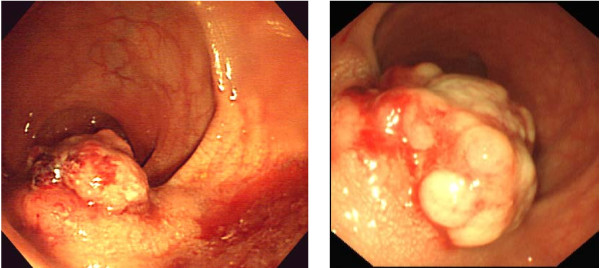
**a) Colonoscopic findings, initial evaluation, b) second evaluation**.

**Figure 2:**
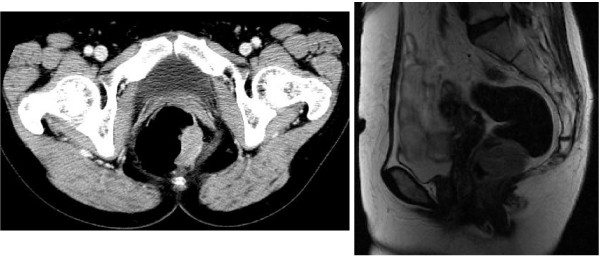
**CT and MRI findings**.

Colonofiberscopy showed an easy-bleeding yellowish tumor with a relatively regular surface with lateral submucosal elevation (Fig. 1a) and 5 biopsied specimens revealed no histological malignancies as in the previous examination. Computed tomography demonstrated a 40 mm diameter tumor on the left side of the lower rectal wall with regional lymphadenopathy. The laboratory data were unremarkable expect for elevated circulating levels of carbohydrate antigen 19-9 (59.1 U/ml; normal value, <37 U/ml). The examinations were repeated 2 and half months later. On colonoscopic examination, the tumor was visualized as more irregular than the previous findings (Fig. 1b) and a biopsied specimen revealed neuroendocrine carcinoma. Computed tomography showed a 50 mm-long tumor in the lower rectum with swollen regional lymph nodes and no distant metastatic lesions (Fig 2).

Based on these findings, the patient underwent abdominoperineal resection with total mesorectal resection and bilateral lymph node dissection. The postoperative course was uneventful. To prevent intrapelvic recurrence, a total dose of 46 Gy in 2 Gy fractions of radiotherapy was delivered through a linear accelerator using the 3-field technique (10 MV), 5 times a week. A daily dose of 800 mg of oral doxifluridine was administered for 5 years because of patient's rejection to intensive intravenous chemotherapy. At 5 years post-surgery, the patient demonstrated no clinical evidence of intrapelvic recurrence or distant metastases.

## Methods

### Immunohistochemistry

IHC was done on formalin-fixed paraffin-embedded sections, using labeled streptoavidin-biotin-peroxidase and microwave antigen retrieval technique. Mouse monoclonal antibodies against chromogranin A (1:50, Dako Cytomation), MIB-1(anti Ki-67,1:50, Dako Cytomation) and p53 protein(1:50, Dako Cytomation) were used. Goat polyclonal antibodies against hASH1 (human acetate-scute homolog 1,1:100, Santa-Cruz, CA, USA) and NeuroD(1:400, Santa-Cruz, CA, USA) were used in order to assess the neuroendocrine differentiation at a transcription level. Mouse IgG was used as a negative control, with dilution of 1:100. Appropriate positive controls known to contain the antigens in question were processed simultaneously.

## Pathological findings

Macroscopically, the resected specimen showed a protruding lesion with an irregular surface, 35 × 20 mm in diameter. The tumor had lateral submucosal elevation and tumor size including lateral elevation was 60 × 30 mm. Microscopically, the tumor invaded the adjacent adipose tissue. Nine of 11 lymph node metastases were observed.

### Immunohistochemistry(IHC)

**Figure 3:**
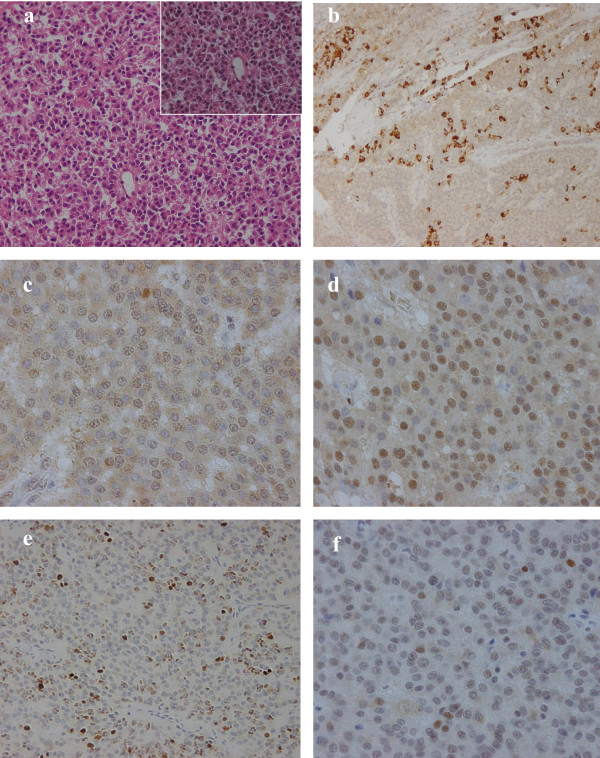
**Pathological findings**. HE staining and Immunohistochemistry pictures: a; HE staining of the tumor (×200), inset a magnification of ×400, b; IHC of chromograninA, which shows strong and diffuse staining in the tumor cytoplasms (×200) c; IHC of hASH1, which shows diffuse staining in the tumor cytoplasms and also in some nucleus (×400) d; IHC of NeuroD, which shows strong and diffuse staining in the tumor nucleus (×400) e; IHC of MIB-1, which shows a high labeling index (×200) f; IHC of p53, which shows partial staining in the tumor nucleus (×400).

ChromograninA showed a diffuse and strong staning in the tumor cytoplasm indicating neuroendocrine differentiation. MIB-1(Ki-67 antigen) labeling index showed 18.3 ± 5.6 supporting high proliferation of the tumor. Nuclear staining of p53 was also detected in approximately 10% of the tumor suggesting the tumor to be an endocrine cell carcinoma. Strong and diffuse nuclear staining of NeuroD and cytoplasmic staining (also in some nucleus) of hASH1 were also detected (Fig. 3).

## Discussion

Neuroendocrine tumors of the colon and rectum represent a broad clinical-pathologic spectrum with varying morphologic features and biological behavior, and there is still much debate concerning their classification. Based on the WHO classification, neuroendocrine tumor of the gastrointestinal tract is classified into 3 subtypes: carcinoid, which is benign or low-grade malignant; malignant carcinoid, which is low-grade malignant; and poorly differentiated neuroendocrine carcinoma, which is high-grade malignant. Poorly differentiated neuroendocrine carcinoma is defined as small cell carcinoma, being morphologically similar to small cell carcinoma of the lung[4]. In addition to small cell carcinoma, pathological studies have shown that moderately differentiated, also known as large cell or intermediate variant, neuroendocrine carcinoma should be classified as high-grade malignant because of its distinct neuroendocrine lineage and biological aggressiveness[1,4]. Moderately differentiated neuroendocrine carcinomas are distinguished from small cell carcinomas by having more vesicular nuclei, more prominent nucleoi, more abundant cytoplasm, and less mitotic activity, morphologically reminiscent of large cell neuroendocrine carcinoma encountered in the lungs. Ki-67 antigen labeling index of the present patient showed 18.3 ± 5.6 supporting high proliferation of the tumor. Ki-67 is expressed by proliferating cells and provides a measurement of the growth fraction in individual tissues and tumors. Some studies suggest that a relationship exists between a high proliferative rate, as measured by Ki-67 immunoreactivity, and tumor aggressiveness[5]. Chaudhry et al. demonstrated that patients with gastrointestinal neuroendocrine tumors with a low Ki-67 index have a better prognosis than tumors with a high proliferative index[6]. According to histopathological findings, our case would be classified as moderately differentiated neuroendocrine carcinoma.

Bernick et al. reported that colorectal moderately differentiated neuroendocrine carcinomas have a poor prognosis with a median survival of only 10.4 months, similar to small cell carcinoma[1]. Patients with neuroendocrine cell carcinoma have liver and lymph node involvement of between 65% and 80% at the time of diagnosis[1,7], therefore, they may benefit from treatment with chemotherapeutic agents. Iyoda et al. showed that adjuvant chemotherapy based on cisplatin, carboplatin, or cyclophosphomide prolongs the survival of patients with large cell carcinoma with neuroendocrine features only in the early stages[8]. In this report, the patient was eager to receive oral but not intravenous chemotherapy. The addition of adjuvant radiotherapy to the primary treatment of rectal cancer has led to the decreased incidence of local recurrence in several randomized studies[9], and radiotherapy was therefore offered postoperatively. Neoadjuvant chemoradiation is considered as a beneficial option, however, surgery was performed by patient's preference.

5-fluorouracil (5-FU) is a key agent that is widely used in the treatment of colorectal cancers. TS is an essential DNA synthetic enzyme that catalyzes the methylation of dUMP to dTMP[10]. DPD is a rate-limiting enzyme of 5-FU catabolism, 85% of an administered dose of 5-FU is degraded to inactive metabolites by DPD[11]. Therefore, low TS and low DPD activity is reportedly correlated with high 5-FU chemosensitivity of cancer cells. Doxifluridine was synthesized by Cook et al[12] and is widely used in Japan as a prodrug of 5-FU, thus, the efficacy of doxifluridine is influenced by levels of TS and DPD. This tumor showed scarce staining of TS and negative staining of DPD, which supports the sensitivity to 5-FU.

p21 is a cyclin dependent kinase inhibitor and its expression is a marker of tumor radiosensitivity in patients with rectal cancer[13]. This tumor had positive staining of p21, indicating the sensitivity to radiation.

This report indicated the difficult histological diagnosis of neuroendocrine carcinoma by endoscopic biopsied specimens. The reason for negative biopsies was speculated its submucosal location. Bernick et al. reported that the sensitivity of preoperative colonoscopic biopsy for colorectal neuroendocrine carcinoma was approximately 60%[1]. We recommend the re-biopsy of an adequate thickness of the rectal wall if a malignant tumor is suspected from the clinical findings and radiological examinations.

In conclusion, we experienced a case of advanced neuroendcrine carcinoma of the rectum with relatively favorable prognosis by postoperative adjuvant chemoradiation therapy.

## Consent

Written informed consent was obtained from the patient for publication of this case report and any accompanying images. A copy of the written consent is available for review by the Editor-in-Chief of this journal.

## Competing interests

The authors declare that they have no competing interests.

## Authors' contributions

HN: deta collection, drafting the manuscript. KS: drafting and revising the manuscript, surgical management of the patient. CK: surgical management of the patient and revising the manuscript. TS:pathological review of surgical specimens, preparing histopathological figures. KK: surgical management of the patient and revising the manuscript. KO: surgical management of the patient and revising the manuscript. SK: pathological review of surgical specimens, preparing histopathological figures. HI: pathological review of surgical specimens, preparing histopathological figures. MM: head of the department who supervised all steps of the work. All authors read and approved final manuscript.
